# Two Very Perplexing Cases

**Published:** 1871-04

**Authors:** H. Scott


### TWO VERY PERPLEXING CASES.

BY H. SCOTT.

Miss S., aged about twenty years, is the subject of a very perplexing case. The first right inferior molar had an incipient crown cavity some eight years since. Upon attempting to fill it I found the dentine so sensitive as to make it impossible for me to prepare the cavity. I have twice used the oxy-chloride, with no good result whatever; on one occasion allowing it to remain over a year. The cavity is not larger than an ordinary pin head, and has not visibly increased from the time of using the os-artificial. It is still so sensitive I can do nothing with it. Sometimes she suffers great pain in attempting to masticate on that side, and occasionally has experienced real toothache. The tooth is well formed and otherwise entirely healthy. I fear to use the arsenic. What shall I do? The lady has had a number of teeth filled, with no unusual sensation. Her teeth are above the average for soundness. She is of sanguine bilious temperament and firm health.

Second case: Mr. R., aged over thirty, called to have the second inferior right molar extracted. It was decayed deeply, but promised firmness enough to be taken out. I applied the proper forceps, and the roots parted, the posterior one coming away with little effort. The front root stood above the alveolus, and was very firm. I applied the curved stump forceps, securing a very firm hold, and expected a quick and easy removal; but to my surprise, after a considerable effort, the bone snapped, leaving a sloping stump with the highest point towards the cheek. I applied the patent key, and after using a rotating force I never like to use, there was another snap, this time sloping outwards and dipping below the margin of the alveolus, thereby still further greatly increasing the difficulties to be overcome. The patient now became faint and we desisted. After half an hour I resumed (there was incipient periostitis in the socket) by cutting the gum down a fourth of an inch over the root, and again applying the key with force, this time I brought away a portion of the alveolus and another fragment of the root, and then abandoned the case by requesting Mr. R. to call the next day, if he were still suffering.

He called yesterday, four days after the operation, with an abscess pointing against the integument at the base of the jaw, and which will inevitably open there. Was there anything that could have been done on my part to prevent this termination of the case, that was not done? What would you have done under the circumstances? I thought at the time I could not for my life remove the root without a very considerable surgical operation. Should any one encounter perplexity like this, I beg them not to imagine they have all the trouble there is going on.

